# Effects of a High-Fat Diet and Docosahexaenoic Acid during Pregnancy on Fatty Acid Composition in the Fetal Livers of Mice

**DOI:** 10.3390/nu15214696

**Published:** 2023-11-06

**Authors:** Daniela Álvarez, Macarena Ortiz, Gabriel Valdebenito, Nicolás Crisosto, Bárbara Echiburú, Rodrigo Valenzuela, Alejandra Espinosa, Manuel Maliqueo

**Affiliations:** 1 Laboratory of Endocrinology and Metabolism, Department of Internal Medicine West Division, Universidad de Chile, Santiago 8350499, Chile; danialvareza@gmail.com (D.Á.); macapaz.ortiz@gmail.com (M.O.); valdebenito@molbio.mgh.harvard.edu (G.V.); nicolascrisostok@gmail.com (N.C.); bechiburu@uchile.cl (B.E.); 2 Endocrinology Unit, Department of Medicine, Clínica Alemana de Santiago, Faculty of Medicine, Universidad del Desarrollo, Santiago 7610658, Chile; 3 Nutrition Department, School of Medicine, Universidad de Chile, Santiago 8380000, Chile; rvalenzuelab@uchile.cl; 4 Department of Medical Technology, Faculty of Medicine, University of Chile, Santiago 8380453, Chile; ealejand@uchile.cl

**Keywords:** pregnancy, high-fat diet, docosahexaenoic acid, maternal liver, placenta, fetal liver

## Abstract

A high-fat diet (HFD) during pregnancy promotes fat accumulation and reduces docosahexaenoic acid (DHA) levels in the liver of the offspring at postnatal ages, which can depend on fetal sex. However, the prenatal mechanisms behind these associations are still unclear. Thus, we analyzed if an HFD alters DHA content and the expression of molecules related to fatty acid (FA) metabolism in the fetal liver. Female C57BL/6 mice were fed a control diet or HFD for 4–6 weeks before pregnancy until the gestational day (GD) 17.5. A subgroup of each diet received DHA (100 mg/Kg) orally from GD 6.5 until 16.5. On GD 17.5, maternal livers, placentas, and livers from male and female fetuses were collected for FA profiling with gas-chromatography and gene expression of molecules related to FA metabolism using qPCR. PPAR-α protein expression was evaluated using Western blot. The gene expression of placental FA transporters was also assessed. An HFD increased eicosapentaenoic acid (EPA) and decreased DHA levels and protein expression of PPAR-α in the fetal livers of both sexes. DHA increased the gene expression of *Ppara*, *Cpt1*, and *Acsl1* in the livers of female fetuses. Therefore, an HFD reduces DHA levels and PPAR-α, a master regulator of gene expression, in the fetal liver. In turn, the livers of female fetuses seem to be more sensitive to DHA action.

## 1. Introduction

Independent of glucose levels, maternal lipids contribute to excess fetal fat accretion and adiposity at birth in pregnancies involving people with obesity [1]. Moreover, there is evidence that a high maternal body mass index is related to lipid accretion in the livers of newborns, suggesting an early start in the events involving the genesis of non-alcoholic fatty liver disease (NAFLD) [2]. This condition is considered the hepatic manifestation of metabolic syndrome, increasing the risk of type 2 diabetes and cardiovascular diseases threefold and eightfold, respectively [3]. Its origin involves multiple hits, starting with hepatic lipid accumulation that can lead to hepatocellular damage and, finally, to hepatic failure, cirrhosis, and cancer [4]. Sex dimorphism seems to be an important factor for the predisposition to NAFLD being more prevalent in males than females in childhood and adulthood [5,6].

Experimental animal models have shown that gestational obesity and obesogenic diets promote hepatic fat accumulation, affecting lipid metabolic pathways, such as de novo lipogenesis and β-oxidation (FAO), pointing out that alterations in maternal metabolism and fetal bioavailability of fatty acids (FAs) contribute to the development of metabolic disorders in the offspring [7,8,9]. In adults, it has been observed that hepatic peroxisome proliferator-activated receptor-alpha (PPAR-α) plays a central role in maintaining hepatic lipid metabolism homeostasis because it regulates the expression of thousands of genes involved in lipogenesis, such as the sterol regulatory element binding protein 1c (*Srebf1*) transcriptional factor, acetyl-CoA carboxylase (*Acaca*), and fatty acid synthase (*Fasn*), in addition to genes related to FAO, including long-chain acyl-CoA synthetase (*Acsl*), carnitine palmitoyltransferase 1 (*Cpt1*), and acyl-CoA oxidase (*Acox*) [10]. Interestingly, in adults, it has been observed that the depletion of *n*-3-PUFA may alter the activation of peroxisome proliferator-activated receptor-alpha (PPAR-α), determining an imbalance in fatty acid β-oxidation in favor of its accumulation in the liver [11].

In the fetal liver, it has been described that low CPT-1 activity and scarce β-oxidation make it susceptible to lipid excess [12]. Interestingly, gestational obesity induced by a saturated-fat-rich diet decreased gene expression of *Cpt1* and *Acox* in the livers of male rat fetuses [13]. On the other hand, the consumption of a high-fat diet (HFD) enriched with dietary *n*-3 polyunsaturated fatty acids (PUFAs), such as α-linolenic acid (C18:3*n*-3, ALA), eicosapentaenoic acid (C20:5*n*-3, EPA), and docosahexaenoic acid (C22:6*n*-3, DHA) in dams, leads to a reduction in lipid accumulation in the fetal liver, suggesting that the composition of the maternal diet is fundamental in the prevention or worsening of this condition [14].

Long-chain PUFAs (LCPUFAs), such as arachidonic acid (C20:4*n*-6, AA) and DHA, are critical for fetal growth during pregnancy since they participate in the formation and maturation of the brain and other organs during fetal development. In this regard, the supply of FA to the fetus depends entirely on maternal consumption, placental transport, and metabolism [15]. The maternal liver is central for understanding fetal FA bioavailability because it synthesizes triglycerides that are packaged into VLDL, which bind to receptors in the placental membranes and are transferred to the fetus [16,17,18,19]. Both obesity during pregnancy and rodent animal models of maternal obesity induced by an HFD show increased placental expression of proteins associated with FA transport and metabolism, suggesting an abnormal ability to transfer FA [18,19].

Therefore, we hypothesize that an obesogenic diet can alter the FA content, mainly DHA, and reduce the protein expression of PPAR-α, affecting the gene expression of molecules related to lipid metabolism in the fetal liver. Then, we analyzed the modifications induced by an HFD on FA composition in the maternal liver, placenta, and fetal liver from male and female offspring. In addition, we explored if the DHA content is associated with the expression of PPAR-α in fetal livers.

## 2. Materials and Methods

Animals: Thirty-two eight-week-old C57BL/6 female mice were fed with a control diet (CD, RMH 3000 LabDiet, containing 15% of kilocalories (Kcal) in fat, 59% of Kcal in carbohydrates, and 26% of Kcal in protein) or HFD (D12341, ResearchDiet, containing 45% of Kcal in fat, 35% of Kcal in carbohydrate, and 20% of Kcal in proteins). The animals were kept in the Central Bioterium of the Faculty of Medicine at the University of Chile in a temperature-controlled room at 20 ± 1 °C, in 12 h light and 12 h dark cycles, with access to water and food ad libitum. The Institutional Ethics Committee at the University of Chile (CICUA) approved all protocols for the care and use of animals (Protocol CBA-1040).

**Figure 1:**
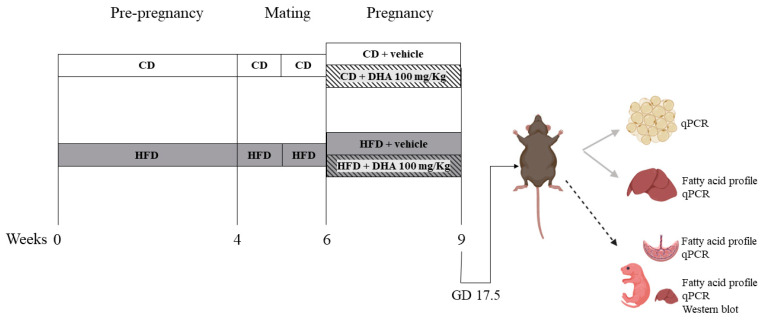
Study design. CD: control diet; HFD: high-fat diet.

Experimental design (Figure 1): Female mice were fed the respective diets for 4–6 weeks until HFD animals gained 20% of their initial body weight. Then, the estrous cycle was monitored via vaginal smears daily. On the morning of the proestrus phase, female mice were placed with male mice with proven fertility. The next morning was considered as gestational day (GD) 0.5. The body weight and food intake were recorded weekly. From GD 6.5 until GD 17.5, a subgroup of CD and HFD dams was supplemented orally with DHA (D2534, Sigma Aldrich, St. Louis, MO, USA) at a dose of 100 mg/Kg/day dissolved in sunflower oil (sc-215936, Santa Cruz Biotechnology, Dallas, TX, USA) as the vehicle conforming CD-DHA (*n* = 7) and HFD- DHA (*n* = 7) groups, respectively. Control groups received isovolumetric amounts of sunflower oil conforming CD-vehicle (*n* = 6) and HFD-vehicle (*n* = 8). At GD 17.5, an oral glucose tolerance test was performed in all dams. Then, they were anesthetized with isoflurane and euthanized via cardiac puncture. Fetuses and placentas were obtained via laparotomy, dried, and weighed. Then, the fetal liver was removed and weighed. In total, forty-two (15 females and 27 males) fetuses were obtained in CD-vehicle group, 44 (25 females and 19 males) in CD-DHA group, 50 (20 females and 30 males) in HFD-vehicle group, and 46 (23 females and 23 males) in HFD-DHA group. All analyses were performed in one or two fetuses per sex in each litter to avoid the litter effect. Maternal liver and adipose tissues were also collected and weighed. Each organ was snap-frozen in liquid nitrogen and stored at −80 °C. Maternal blood was centrifuged at 10,000 rpm for 10 min to obtain serum.

Oral glucose tolerance test (OGTT): after 4 h of fasting, a basal blood sample was taken by cutting the tip of the tail to measure glycemia with a glucometer (OneTouch^®^ UltraMini^®^, Johnson & Johnson, New York, NY, USA) and insulin. A glucose solution dissolved in water (2 g/kg) was then administered orally by gavage, and glycemia was measured at 15, 30, 60, and 90 min. At basal and after 15 min of glucose administration, 30 μL of blood was taken, centrifugated at 10,000 rpm for 10 min at 4 °C, and the plasma was separated for insulin measurement. In the basal sample, the Homeostatic Model Assessment for Insulin Resistance (HOMA-IR) was determined as follows: HOMA-IR = [Insulin (U/L) × Blood glucose (mmol/L)]/22.5 [20].

Genotyping of fetal sex: The tail of each fetus was placed in 250 μL of 50 mM NaOH and heated at 98 °C for 30 min. Then, 125 μL of the solution was mixed with the same volume of distilled water and 25 μL of 1 M Tris-HCL pH 8.0. Then, 5 μL was used to identify the *Sry* gene with PCR and resolved in a TAE gel at 2%.

Lipid profile and insulin: Triglyceride, total cholesterol, and high-density cholesterol (HDL) levels were measured using an enzymatic method (Biosystem, Barcelona, Spain). Insulin concentrations were determined using enzyme-linked immunoassays according to the manufacturer’s instructions (10-1247-10, Mercodia Mouse Insulin ELISA, Uppsala, Sweden). The intra-assay coefficient of variation was less than 2.0% for the lipid profile and 5.0% for insulin. The limit of detection for insulin was 200 pg/mL. 

Fatty acid profile: Quantitative extraction and separation of total lipids was carried out according to Bligh and Dyer [21]. Briefly, liver (200 mg for dams and 100 mg for fetuses) and placenta (100 mg) samples were homogenized with 1 mL of internal standard (methyl tricosanoate, C23; Nu-Chek Prep Inc., Elysian MN, USA), 2 mL of chloroform, and 2 mL of methanol. Sodium biphosphate 0.2 M was added and centrifuged (3000 rpm × 10 min) to collect the lipid phase. The saponifiable lipids were derivatized to methyl esters via alkaline hydrolysis (NaOH saturated in methanol 0.5 M) and then acidified with BF_3_ (12% in methanol). The FA profile was performed using gas–liquid chromatography (7890A, Agilent Technologies, Santa Clara, CA, USA) with a capillary column (HP-88, 100 MX 0.250 mm; ID 0.25 um, Agilent Technologies, Santa Clara, CA, USA). Values are expressed as mg per 100 g FAME. 

RNA isolation, cDNA synthesis, and quantitative PCR: In total, 25 mg of the maternal and fetal livers, placenta, or adipose tissue was homogenized in 1 mL of TRI Reagent (T9424, Sigma-Aldrich, St. Louis, MO, USA). RNA was isolated with E.Z.N.A Total RNA kit with DNAse (OBR6834-02CH and OBE1091-02, Omega-Bio-Tek, Norcross, GA, USA) according to the manufacturer’s instructions. The cDNA was synthesized from 1 μg of RNA using a High-Capacity cDNA Reverse Transcription Kit (4,368,814, ThermoFisher Scientific, Waltham, MA, USA). Real-time quantitative PCR was performed using Fast SYBR^®^ Green PCR Master Mix (4,385,612, Applied Biosystems, Waltham, MA, USA) in AriaMx Real-time PCR System (Agilent Technologies, Santa Clara, CA, USA). Expression levels were determined using the Pfaffl method with normalization to *Rpl30* and *Pplp0* expression. Sequences of specific primers are shown in Supplementary Table S1.

Western blot analyses: Proteins from fetal liver samples (25 mg) were isolated in radioimmunoprecipitation assay (RIPA) buffer (R0278, Sigma-Aldrich, St. Louis, MO, USA) containing protease inhibitor cocktail (P8340, Sigma-Aldrich, St. Louis, MO, USA), orthovanadate, and phenylmethylsulfonyl fluoride (PMSF). Samples were centrifuged at 10,000× *g* for 10 min. Twenty micrograms of total protein was separated on 12% polyacrylamide gels under reducing conditions. Proteins were transferred to a nitrocellulose membrane, blocked with 5% milk for 1 h, and probed overnight with the primary antibody PPAR-α (1:2000, SC-398394, Santa Cruz Biotechnology, Dallas, TX, USA). Protein bands were developed with Clarity^TM^ Western ECL Substrate (Bio-Rad Laboratories, Hercules, CA, USA) and photographed with ChemiDoc XRS+System (Bio-Rad Laboratories, Hercules, CA, USA). Results were analyzed by measuring the pixel intensities of bands using the Image Lab 6.0 (Bio-Rad Laboratories, Hercules, CA, USA) program. Relative protein levels were calculated using β-actin (1:10,000, SC-69879, Santa Cruz Biotechnology, Dallas, TX, USA) as an internal control. All Western blots were performed in duplicate.

Statistical analysis: Data are expressed as mean ± standard error of the mean (SEM). Two-way ANOVA following the Sidak post-test was used to test the effects of diet and DHA treatments. The effects of the diet and treatments on food intake and body weight before and during pregnancy were analyzed with repeated-measures ANOVA. The association between PPAR-α protein expression and DHA content in the fetal liver was evaluated with Pearson’s correlation test. All analyses of the offspring were performed separately for males and females. Statistical analysis was performed with GraphPad Prism version 9.4.1 (GraphPad Software, San Diego, CA, USA). *p* < 0.05 was considered statistically significant.

## 3. Results

### 3.1. Maternal Characterization

To characterize the effects of an HFD and DHA administration on maternal weight gain and metabolic parameters, we evaluated weight gain, food intake, weight of fat depots and liver, circulating glucose and insulin levels, lipid profile, and liver lipid content. 

#### 3.1.1. Biometrics Parameters and Food Intake

**Figure 2:**
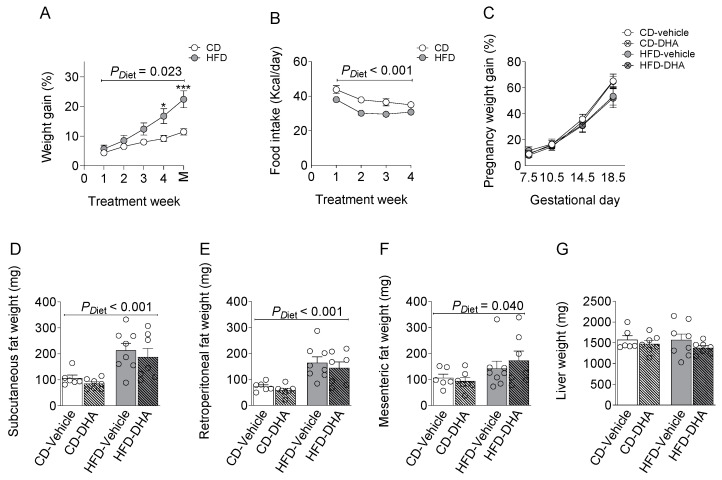
Characterization of dams fed with a control diet (CD) or high-fat diet (HFD) and treated with vehicle (sunflower oil) or docosahexaenoic acid (DHA) during pregnancy. (**A**,**B**) Pregestational weight gain and food intake; (**C**) gestational weight gain; (**D**–**F**) subcutaneous, retroperitoneal, and mesenteric fat depots; (**G**) maternal liver weight. Values are means ± SEM. Two-way ANOVA followed by Sidak’s post-test was performed to calculate the differences. *** *p* < 0.001 and * *p* < 0.05 between CD and HFD. M = mating.

From 4 weeks of the diet, dams in the HFD group gained more weight than controls, although the food intake was lower at 4 weeks of the diet (Figure 2A,B). All dams increased their body weight during the gestational period without differences due to diet or DHA supplementation (Figure 2C). The HFD increased the weight of subcutaneous, mesenteric, and retroperitoneal fat depots compared with groups fed with the CD (Figure 2D–F). After normalization by body weight, fat depots remain higher in the HFD rather than the CD (Supplementary Table S2). On the other hand, no changes were observed in maternal liver weights (Figure 2G). DHA administration did not induce changes in any biometric parameters. 

#### 3.1.2. Metabolic Parameters

**Figure 3:**
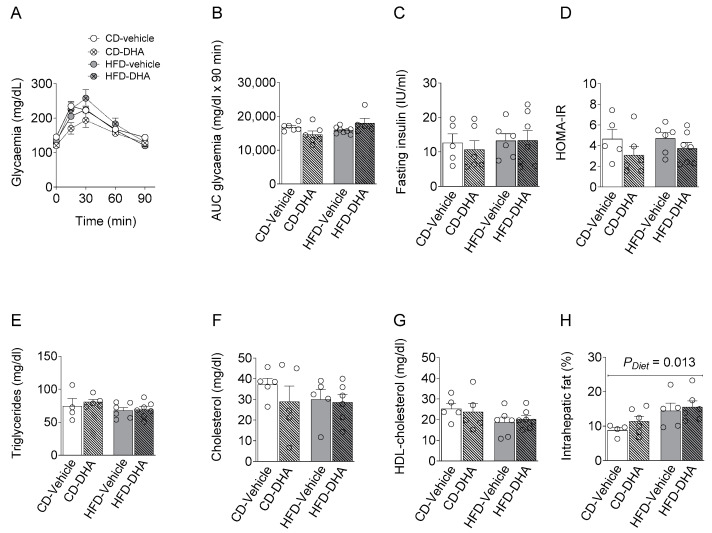
Maternal metabolic parameters at gestational day (DG) 17.5. (**A**,**B**) Glucose levels and area under the curve (AUC) during an oral glucose tolerance test (OGGT); (**C**) fasting insulin serum concentration; (**D**) homeostatic model assessment (HOMA-IR); (**E**–**G**) fasting triglycerides, cholesterol and HDL; (**H**) percentage of intrahepatic fat content. Values are means ± SEM. Two-way ANOVA followed by Sidak’s post-test was performed to calculate the differences.

There were no differences between groups in maternal serum levels of basal glycemia during the oral glucose tolerance test (OGTT) or its area under the curve, basal serum insulin levels, and HOMA-IR (Figure 3A–D). Similar observations were found in the lipid profile (Figure 3E–G). On the other hand, a main effect of diet was observed in the intrahepatic fat content (Figure 3H).

### 3.2. Fetal Biometry Characterization

To evaluate the effects of an HFD and DHA treatments on the fetal growth of male and female fetuses, biometric parameters, including placental, fetal body weight, and liver parameters, were measured. Moreover, placental efficiency was calculated as fetal and placental weight ratio.

**Figure 4:**
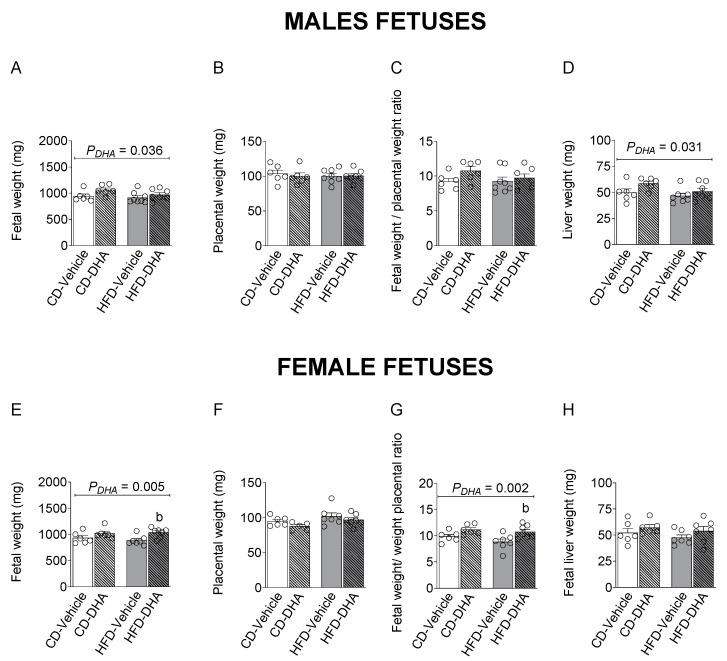
Biometric measurements in fetuses from dams fed with a control diet (CD) or high-fat diet (HFD) and treated with vehicle (sunflower oil) or docosahexaenoic acid (DHA) during pregnancy. Fetal and placental weight, fetal–placental weight ratio, liver weight, and liver–body weight ratio in male (**A**–**D**) and female fetuses (**E**–**H**). Values are means ± SEM. Two-way ANOVA followed by Sidak’s post-test was performed to calculate the differences. ^b^ *p* < 0.05 between HFD-vehicle and HFD-DHA groups.

Fetal and placental weight, the fetal–placental weight ratio, and liver weight were not affected by the diet in both sexes (Figure 4A–H). However, the HFD decreased the liver–fetal weight ratio in females (*p* = 0.017). On the other hand, DHA increased fetal and liver weight in males (Figure 4A,D). In females, a main effect of DHA was observed in fetal weight and the fetal–placental weight ratio, which were increased in the group with the HFD (*p* = 0.012 and *p* = 0.016, respectively) (Figure 4A,G).

### 3.3. Fatty Acid Composition

The maternal liver and placenta are central in the FA transfer to the fetus, and the fetal liver receives a considerable part of the nutrients transported by the placenta; we determined the FA profiling, including saturated FA (SFA), monounsaturated FA (MUFA), *n*-3 PUFA, and *n*-6 PUFA in the maternal liver and placenta.

#### 3.3.1. Maternal Liver Fatty Acid Composition

A main effect of the diet was observed in the distribution of the different SFAs, MUFAs, *n*-3 PUFAs, and *n*-6 PUFAs (Supplementary Table S3). In this regard, the HFD increased the amount of lauric acid (C12:0), myristic acid (C14:0), palmitic acid (C16:0), and total SFA. The concentrations of oleic acid (C18:1*n*-9, OA), eicosanoic acid (C20:1), and total MUFA were also higher in dams fed with the HFD than in the CD. Similarly, the HFD increased linoleic acid (C18:2*n*-6, LA), eicosadienoic acid (C20:2*n*-6), the gamma-linolenic acid (C18:3*n*-6, GLA) to LA ratio, and total *n*-6 PUFA concentration, but it reduced docosapentaenoic acid (C22:5*n*-3, *n*-3 DPA) and the *n*-3 to *n*-6 PUFA ratio. Finally, DHA supplementation did not modify the concentration of any SFA, MUFA, or *n*-6 and *n*-3 PUFA. An interaction between HFD and DHA was observed in the EPA to ALA ratio, which was increased in the group with the HFD (*p* = 0.017) (Supplementary Table S3).

#### 3.3.2. Placenta Fatty Acid Composition

**Figure 5:**
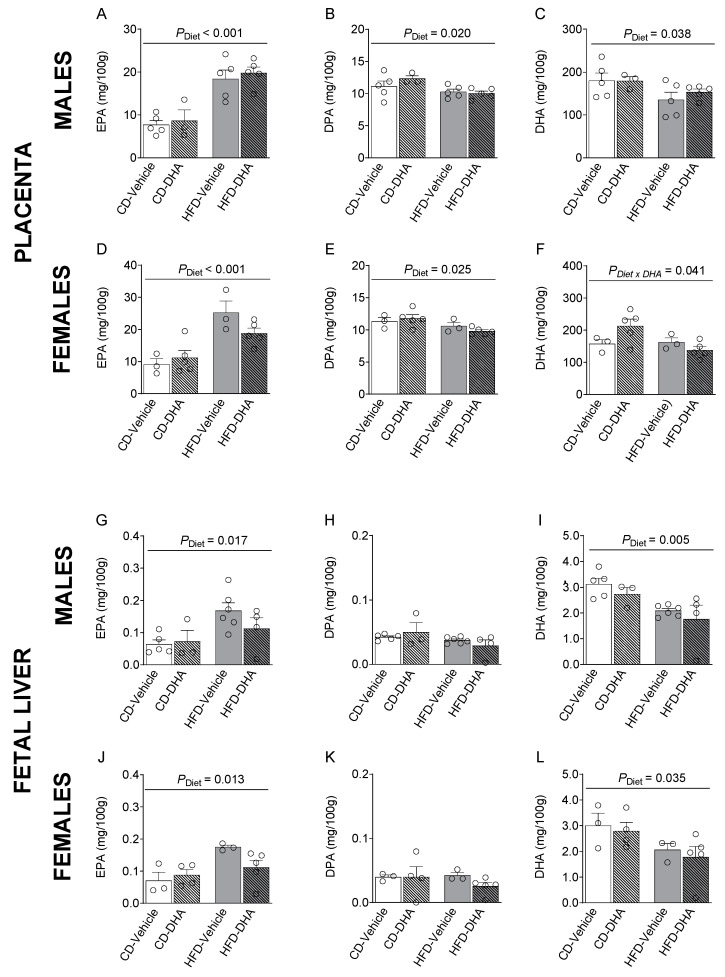
Composition of *n*-3 polyunsaturated fatty acids in the placentas and livers of male and female fetuses from dams fed with a control diet (CD), high-fat diet (HFD), and treated with vehicle (sunflower oil) or docosahexaenoic acid (DHA) during pregnancy. Levels of eicosapentaenoic acid (C20:5 *n*-3, EPA), docosapentaenoic acid (C22:5 *n*-3, DPA), and docosahexaenoic acid (C22:6 *n*-3, DHA) in placentas (**A**–**F**) and fetal livers (**G**–**L**). Values are means ± SEM. Two-way ANOVA followed by Sidak’s post-test was performed to calculate the differences.

In placentas from male and female fetuses, a main effect of the HFD was observed in SFAs and MUFAs with lower levels of lignoceric acid (C24:0) and erucic acid (C22:1 *n*-9) in the HFD compared with the CD (Supplementary Tables S4 and S5). Moreover, in females, the main effect of DHA treatment was an increased palmitoleic acid (C16:1 *n*-7), whereas in males, DHA treatment increased dihomo-γ-linolenic acid (C20:3*n*-6, DGLA), mainly in the HFD group (*p* < 0.001, Supplementary Table S4). In placentas from female fetuses, an interaction between HFD and DHA was observed on lignoceric acid, erucic acid, AA, and total *n*-6 PUFA levels (Supplementary Table S5). Moreover, the main diet effects were found in *n*-3 PUFA levels, of which EPA was higher in fetuses of both sexes from the HFD compared with the CD (Figure 5A,D). Then, the EPA to ALA ratio was also higher in the HFD group (Supplementary Tables S4 and S5). On the other hand, DPA was lower in the HFD compared with the CD (Figure 5B,E). DHA was lower in males and tended to be lower in females (*p* = 0.072) (Figure 5C,F). In females, an interaction between diet and DHA treatment was observed in DHA levels (Figure 5F). Similar observations were found in ALA, total *n*-3 PUFA, and total PUFA. A main effect of diet was also observed in the *n*-3/*n*-6 ratio in females in the HFD compared with the CD (Supplementary Table S5).

#### 3.3.3. Fetal Liver Fatty Acid Composition

In the male liver, the main effects of the diet were observed in the levels of lignoceric acid, GLA, and the GLA to LA ratio, which was lower in fetuses from dams fed with the HFD than the CD (Supplementary Table S6). In female fetuses, we found a main effect of diet with lower levels of erucic acid in the HFD compared with the CD (Supplementary Table S7). Also, DHA had a main effect in male fetuses, with lower levels of behenic acid (C22:0). In this regard, in the HFD group, palmitoleic acid and total MUFA were lower in fetuses from mothers that received DHA compared with those that did not receive it. An interaction between diet and DHA was observed in OA and total MUFA (Supplementary Table S7). Interestingly, the HFD had a main effect on EPA, which was higher in the HFD than in the CD in both male and female fetuses (Figure 5G–J). However, DHA was lower in fetuses of both sexes from dams fed with the HFD (Figure 5I,L). Moreover, in males, total PUFA and *n*-3 PUFA were lower in the HFD than in the CD (Supplementary Table S6).

### 3.4. Gene Expression

Pro-inflammatory environments have been associated with abnormal maternal–fetal FA transfer [22]. Then, we analyzed the mRNA expression of pro-inflammatory cytokines, including tumoral necrosis factor alpha (Tnfa), interleukin-6 (Il6), and monocyte chemoattractant protein-1 (Mcp1), in the maternal adipose tissue and liver to test if an HFD and DHA administration could explain the changes observed in FA composition in fetal livers. Moreover, we tested in the placenta the gene expression of endothelial lipase (Lipg) because of its role in the hydrolysis of triglycerides to release FAs to be transported, the fatty acid transporter 4 (Scl27a4) and fatty acid translocase (Cd36), due to their participation in FA transport; in addition, we included major facilitator superfamily domain containing 2A (Mfsd2a) given its relevance and specificity in the transport of DHA [23]. In the fetal liver, we assessed the expression of genes encoding for proteins related to regulators of FA metabolism, such as peroxisome proliferator-activated receptor alpha (Ppara) and acetyl-CoA carboxylase alpha (Acaca); genes associated with lipogenesis, such as sterol regulatory element binding transcription factor (Srebf), acyl-CoA oxidase (Acox), and fatty acid synthase (Fasn); and those related to FAO, such as carnitine palmitoyltransferase I (Cpt1), long-chain acyl-CoA synthetase 1 (Acsl1), and acyl-CoA oxidase (Acox) [24]. Moreover, 5-lipoxygenase (5-Lox) participates in the biosynthesis of pro-resolving lipid mediators derived from DHA [25].

**Figure 6:**
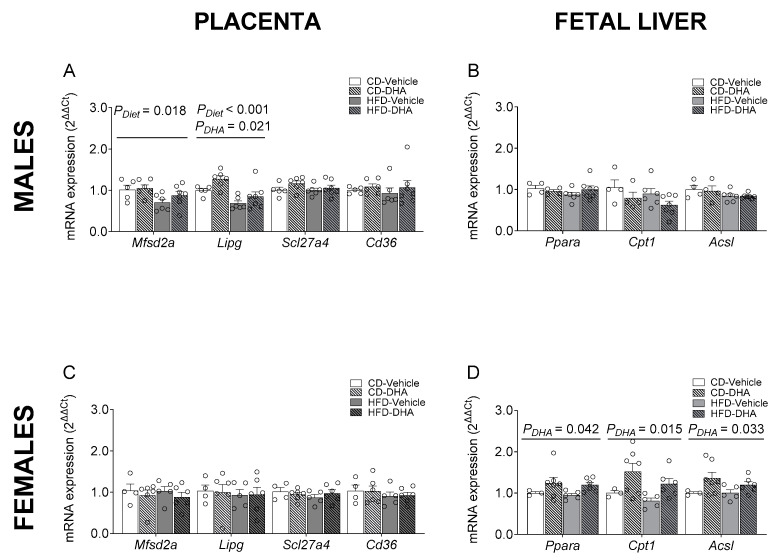
Gene expression of placental fatty acid transporters and regulators of fatty acid metabolism in the fetal livers of male and female fetuses from dams fed with a control diet (CD), high-fat diet (HFD), and treated with vehicle (sunflower oil) or docosahexaenoic acid (DHA) during pregnancy. Gene expression of *Mfsd2a*, *Lipg*, *Scl27a4*, and *Cd36* in placentas from male and female fetuses (**A**,**C**). Gene expression of *Ppara*, *Cpt1*, and *Acsl1* in the livers of male and female fetuses (**B**,**D**). Values are means ± SEM. Two-way ANOVA followed by Sidak’s post-test was performed to calculate the differences.

The gene expression of proinflammatory cytokines such as Il6, Tnfa, and Mcp1 was not different between groups in the maternal liver (Supplementary Figure S1A). Similar results were observed in maternal adipose tissue, except for the expression of Mcp1, which had an interaction effect between diet and DHA, with lower levels in DHA compared with vehicle in the HFD group (Supplementary Figure S1B). In placentas from male fetuses, the HFD showed a main effect of diet that caused a decrease in the mRNA expression of Mfsd2a and Lipg. Moreover, a main effect of DHA treatment was observed in the expression of Lipg (Figure 6A). The gene expressions of Scl27a4, Cd36, Cpt1, Ppara, Il-6, Tnfa, and Mcp1 were similar between groups (Supplementary Figure S1C). On the other hand, in placentas from female fetuses, no differences were found in gene expression (Figure 6C and Supplementary Figure S1D). No effects of diet or DHA treatment were observed in the mRNA expression of lipogenic and FAO enzymes in male fetal livers (Figure 6B and Supplementary Figure S1E). In contrast, in female fetuses, a main effect of DHA treatment with higher levels of Ppara, Cpt1, and Acsl1 was found (Figure 5D). No effects of diet or DHA treatment were observed in the mRNA expression of 5-Lox in male and female fetal livers (Supplementary Figure S1E,F).

### 3.5. Protein Expression

Finally, we tested the protein expression of transcription factor PPAR-α, a master regulator of the expression of genes associated with lipogenesis and FAO; moreover, it is regulated by DHA [26]. 

**Figure 7:**
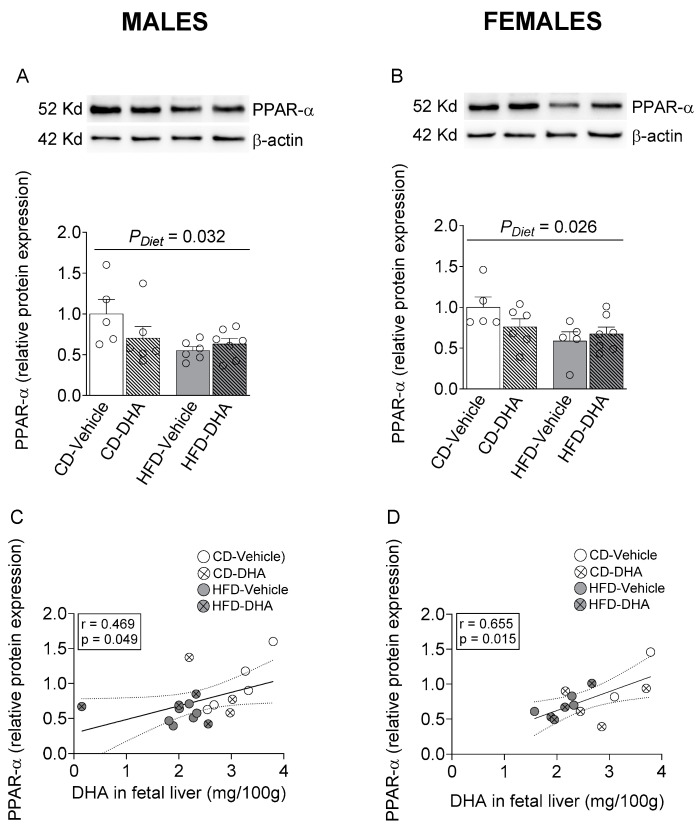
Protein expression of peroxisome proliferator-activated receptor alpha (PPAR-α) in the fetal livers of male and female fetuses from dams fed with a control diet (CD), high-fat diet (HFD), and treated with vehicle (sunflower oil) or docosahexaenoic acid (DHA) during pregnancy. Protein expression of PPAR-α in the fetal liver (**A**,**B**). Correlation between protein expression of PPAR-α with the content of DHA in the fetal liver (**C**,**D**). Values are means ± SEM. Two-way ANOVA followed by Sidak’s post-test was performed to calculate the differences. Correlation coefficients were computed using the Pearson correlation test.

In male and female fetal livers, a main effect of diet caused a decreased protein expression of PPAR-α (Figure 7A,B). Interestingly, a positive correlation between PPAR-α and DHA content in the fetal liver was found in both males and females (Figure 7C,D). 

## 4. Discussion

The main findings of this study are that the HFD reduced DHA and increased EPA levels in the livers of male and female fetuses. These changes were associated with lower protein expression of PPAR-α, whereas DHA administration increased the expression of lipid metabolism genes such as *Ppara*, *Acsl1*, and *Cpt1* in female fetuses. On the other hand, the HFD reduced the gene expression of *Lipg* and *Mfsd2a* in placentas from male fetuses but had no difference in placentas from female fetuses. 

Maternal obesity associated with an abnormal profile of FAs, including elevated levels of SFAs and *n*-6 PUFAs, has been associated with alterations in the lipid metabolism in offspring in adulthood, suggesting that the origin of this phenomenon lies in an abnormal maternal–fetal transfer of FA due to alterations in maternal or placental lipid metabolism [27,28,29,30]. In the present study, an HFD increased the maternal intrahepatic fat content with a predominance of the total amount of SFA, MUFA, and *n*-6 PUFA levels, usually observed in subjects with NAFLD. On the other hand, *n*-3 PUFA was not entirely affected by the HFD, showing lower *n*-3 DPA levels and similar levels of DHA. It has been shown that HFD increases elongase activity, which could increase the DHA synthesis rate from *n*-3 DPA [31,32]. Moreover, adipose tissue is the main site for PUFA accretion during pregnancy, and liver DHA is rapidly mobilized to placental uptake, which can explain why the administration of DHA did not increased its content in the maternal liver [33,34]. Therefore, despite the abnormalities in FA composition in the maternal liver, the DHA levels seem unaffected. 

The maternal composition of FAs affects *n*-3 PUFA placental uptake, such as LA reduces ALA and DHA placental content. Interestingly, we observed an increase in LA in the maternal liver, suggesting a high maternal bioavailability of this FA. This phenomenon has been associated with limited placental uptake due to modifications in the expression of placental transporters of *n*-3 fatty acid [35]. Moreover, in placentas from male fetuses from the HFD group, we observed a reduced gene expression of *Mfsd2a*, a membrane lysophospholipids transporter required to uptake DHA in the brain and placenta [36,37]. Interestingly, in the human placenta, one study found a lower gene expression of this transporter in the placenta from male fetuses of women with obesity [38]. In the same way, the HFD downregulated the gene expression of *Lipg*, which is a phospholipase A1 with a limited ability to release sn-2–bound unsaturated FA from phospholipids. Interestingly, DHA transport is preferentially associated with phospholipids over other lipids, so the action of the endothelial lipase enzyme must be hydrolyzed and taken up by the placenta [39]. On the other hand, in females, we did not observe significant HFD-induced changes in FA composition, apart from higher EPA and lower DPA *n*-3 levels, suggesting a dimorphic effect on placental FA uptake or metabolism.

Along with lower DHA levels induced by the HFD, increased levels of EPA indicated a possible alteration in *n*-3 PUFA metabolism, which can be related to increased biosynthesis or decreased degradation. In this regard, it has been observed that DHA can be retroconverted to DPA and then to EPA due to peroxisomal action [40,41]. In the same way, reduced levels of C24:0 (tetracosanoic acid) and C22:1 n-9 (erucic acid) suggest increased FAO in peroxisomes of the placenta from the HFD group [42]. Supporting this concept, in women with obesity, an increase in peroxisomal FAO has been observed [43]. Interestingly, it has been observed that the induction of peroxisomal FAO serves as a mechanism for lipid accumulation in other tissues of obese mice [44]. Therefore, alterations in FA composition suggest that peroxisomal function can be affected by an HFD, reducing DHA bioavailability.

In this way, we cannot rule out that reduced levels of DHA are a consequence of its metabolization to pro-resolving products with anti-inflammatory properties such as resolvin D1 and D2 [45]. Regarding this, no differences were observed in the expression of pro-inflammatory cytokines in the placenta. Concordantly, DHA supplementation increased placental efficiency in female fetuses from dams fed with the HFD, indicating an improvement in the placental function to support fetal growth. 

In general, alterations in placental uptake and metabolism of FAs are related to maternal hyperglycemia, insulin resistance, and a pro-inflammatory state that modifies the expression of placental transporters, lipid metabolism, and storage or impairs mitochondrial β-oxidation that favors peroxisomal oxidation [46]. However, despite dams showing increased body weight and fat mass, we observed no significant changes in glucose levels, insulin resistance, lipid profiles, and mRNA expression of proinflammatory cytokines in adipose tissue, indicating, in our model, that the changes in the placental FA composition result from the lipid overload induced by the HFD rather than from the maternal metabolic disturbances, suggesting the fundamental role of a healthy diet in the placental function independent of the maternal metabolic conditions. In this regard, evidence in humans has shown a reduced materno–fetal transfer of DHA in normolipidemic women with obesity compared with women with target weights [47]. 

The FA pattern in the placentas from both male and female fetuses is very similar to the FA pattern found in the fetal livers, highlighting the regulatory role of the placenta in FA maternal–fetal transfer. In adults, DHA activates PPARα, regulating the expression of thousands of metabolic genes associated with de novo lipogenesis and FAO. In this regard, an HFD leads to reduced levels of DHA, concomitantly with lower PPARα protein expression in the fetal livers of both sexes. However, we did not observe changes in the gene expression of lipogenic or FAO enzymes. However, in the male fetal liver, the ratio of GLA/LA was lower in the HFD group, suggesting a reduced Δ-6 desaturase activity, which has been correlated with markers of oxidative stress and early steps of NAFLD [11]. Relatively few studies have focused on studying fetal hepatic metabolism. However, a study in rats indicates that maternal obesity induced by a diet rich in saturated fat leads to decreased gene expression of *Cpt1* and *Acox* in male fetuses [13], in addition to an increase in AST, ALT, signs of inflammation, and oxidative stress in the fetal liver, both in males and females [48]. 

On the other hand, the administration of DHA reduced palmitoleic acid and total MUFA in the fetal liver with a higher effect in fetuses from dams fed with an HFD, suggesting that DHA could reduce the activity of SCD1 and protect the fetus from fat accumulation and hepatic steatosis, such as has been shown in obese mice models supplemented with fish oil [49,50]. Interestingly, fetal hepatic levels of DHA were positively correlated with the expression of PPAR-α in both sexes. However, DHA only increased the expression of FAO-related genes in female fetuses, suggesting a sex-dependent sensitivity to DHA in these genes. 

Our study has the strength of analyzing the profile of FAs in the maternal, placenta, and fetal compartments, providing a broad landscape of the metabolic adaptations related to FA metabolism established to support fetal development. Moreover, all measurements in the placenta and fetal livers were performed considering the sex dimorphism because of its crucial role in the energetic response induced by dietary challenges [51]. A possible limitation of our study is that the ratio of male to female fetuses was not homogenous in each litter, which limited the number of samples in some measurements. On the other hand, our model did not show maternal metabolic alterations other than an increased fat mass because we aimed to avoid the effects of hyperglycemia and elevated insulin levels on placental function; therefore, this allows us to attribute our findings to dietary effects and maternal adiposity rather than to metabolic disturbances associated with glucose metabolism.

## 5. Conclusions

Our results show that an HFD, independent of abnormalities in maternal glucose metabolism, induced reduced levels of DHA in the livers of both male and female fetuses, which seems to be associated with the altered transport and metabolism of FAs in the placenta, mainly in male fetuses. In turn, DHA administration did not reverse all the HFD-induced effects but reduced the levels of MUFAs in male fetuses. In contrast, in female fetuses, DHA increased the expression of genes associated with regulating lipid metabolism, such as *Ppara*, *Cpt1*, and *Acsl1*, suggesting a possible protective role against hepatic lipid accumulation in both males and females.

## Data Availability

Data are contained within the article and supplementary materials.

## Supplementary Materials

The following supporting information can be downloaded at: https://www.mdpi.com/article/10.3390/nu15214696/s1, Supplementary Table S1: Primers sequences; Table S2: Maternal fat depots and liver weight normalized by total body weight; Table S3: Fatty acid profile in maternal livers; Table S4: Fatty acid profile in placentas from male fetuses; Table S5: Fatty acid profile in placentas from female fetuses; Table S6: Fatty acid profile in liver from male fetuses; Table S7: Fatty acid profile in liver from female fetuses; Figure S1: Gene expression (mRNA) in maternal tissues, placentas, and fetal livers of male and female fetuses.
