# Supplementary material for: Effects of a High-Fat Diet and Docosahexaenoic Acid during Pregnancy on Fatty Acid Composition in the Fetal Livers of Mice
Source: Nutrients. 2023 Nov 6;15(21):4696. doi: 10.3390/nu15214696 (PMC10649644; doi:10.3390/nu15214696)
Supplement: Supplementary file 1 [file nutrients-15-04696-s001.zip › nutrients-2673161-supplementary/Suplemmentary material/Suplemmentary tables.pdf]

Supplemental Table 1. Primers sequences

**Gen**	**Protein name**	**Sequence primers**	**Tm (°C)**	**GC (%)**	**Gene ID**
Mfsd2a	Major facilitator superfamily domain containing 2A	F: 5' - TCTGGTGGGCTTCTGCATTAG - 3'<br>R: 5' - GATGGGAAGTCAGGCACAAAC - 3'	61.3<br>61.3	52.4<br>52.4	76574
Lipg	Endothelial lipase	F: 5' - ATGCGAAACACGGTTTTCCTG - 3'<br>R: 5' - GGACGCAAGGTTGTGATACTTC - 3'	59.4<br>62.1	47.6<br>50.0	16891
Scl27a4	Fatty acid transporter 4	F: 5' - GACTTCTCCAGCCGTTTCCA - 3'<br>R: 5' - AGGACAGGATGCGGCTATTG - 3'	60.5<br>60.5	55.0<br>55.0	26569
Cd36	Fatty acid translocase	F: 5' - GGCTGTGTTTGGAGGCATTC - 3'<br>R: 5' - CCACGTCATCTGGGTTTTGC - 3'	60.5<br>60.5	55.0<br>55.0	12491
Cpt1	Carnitine palmitoyltransferase I	F: 5' - CTCAGTGGGAGCGACTCTTCA - 3'<br>R: 5' - GGCCTCTGTGGTACACGACAA - 3'	63.3<br>63.3	57.1<br>57.1	12894
Acsl1	Long-chain acyl-CoA synthetase 1	F: 5' - TCTTGGTGTACTACTACGACGAT - 3'<br>R: 5' - CGAGAACCTAAACAAGGACCATT - 3'	61.1<br>61.1	43.5<br>43.5	14081
Acox	Acyl-CoA oxidase	F: 5' - TCGAAGCCAGCGTTACGAG - 3'<br>R: 5' - GGTCTGCGATGCCAAATTCC - 3'	59.5<br>60.5	57.9<br>55.0	11430
Acaca	Acetyl-CoA carboxylase alpha	F: 5' - CCTGACAAACGAGTCTGGCT – 3'<br>R: 5' - CATTCCATGCAGTGGTCCCT – 3'	62.2<br>63.1	55.0<br>55.0	107476
Fasn	Fatty acid synthase	F: 5' - GCTGCGGAAACTTCAGGAAAT - 3'<br>R: 5' - AGAGACGTGTCACTCCTGGACTT - 3'	59.4<br>64.7	47.0<br>52.2	14104
Srebf1	Sterol regulatory element binding transcription factor	F: 5' - ACTGGCCGAGATGTGCGAA - 3'<br>R: 5' - AGCATAGGGGGCGTCAAACA - 3'	59.5<br>60.5	57.9<br>55.0	20787
5-Lox	5-lipoxygenase	F: 5' – AGTGACAGGGTCAAGAAGTTGG - 3'<br>R: 5' - GCCCCGTTCGAAGTCATTGT- 3'	60.2<br>61.0	50.0<br>55.0	11689
Ppara	Peroxisome proliferator-activated receptor alpha	F: 5' - CTGGGCAAGAGAATCCACGA - 3'<br>R: 5' - CGTCTTCTCGGCCATACACA - 3'	60.5<br>60.5	55.0<br>55.0	19013
Il-6	Interleukin 6	F: 5' - GAGACTTCCATCCAGTTGCCT - 3'<br>R: 5' - TGGGAGTGGTATCCTCTGTGA - 3'	61.3<br>61.3	52.0<br>52.0	16193
Tnfa	Tumoral necrosis factor alpha	F: 5' - CACCACGCTCTTCTGTCTACT - 3'<br>R: 5' - GGTCTGGGCCATAGAACTGAT - 3'	61.3<br>61.3	52.0<br>52.0	21926
Mcp1	Monocyte chemoattractant protein - 1	F: 5' - CCTGCTGCTACTCATTCACCA - 3'<br>R: 5' - CATTCCTTCTTGGGGTCAGCA - 3'	61.3<br>61.3	52.0<br>52.0	20296
Sry	Testis-determining factor	F: 5' -TTGTCTAGAGAGCATGGAGGGCCATGTCAA - 3'<br>R: 5' - CCACTCCTCTGTGACACTTTAGCCCTCCGA - 3'	68.7<br>70.3	50.0<br>56.6	21674

Supplemental Table 2. Maternal fat depots and liver weight normalized by total body weight.

	CD-vehicle (n = 6)	CD-DHA (n = 7)	HFD-vehicle (n = 8)	HFD-DHA (n = 7)	P_{Diet}	P_{DHA}	$P_{Diet\ x\ DHA}$
Subcutaneous fat/ BW	2.83 ± 0.37	2.46 ± 0.24	5.66 ± 0.73	5.24 ± 0.95	**< 0.001**	0.556	0.974
Mesenteric fat/ BW	2.80 ± 0.36	2.77 ± 0.51	3.66 ± 0.55	7.17 ± 2.24	**0.039**	0.162	0.156
Retroperitoneal/ BW	1.90 ± 0.18	1.69 ± 0.24	4.28 ± 0.42	4.00 ± 0.66	**< 0.001**	0.578	0.945
Liver/ BW	41.40 ± 2.02	42.86 ± 2.69	41.49 ± 3.13	38.29 ± 0.69	0.369	0.725	0.351

Values are means ± SEM. Differences were calculated by two-way ANOVA followed Sidak's post-test. BW: Body weight

Supplemental Table 3. Fatty acid profile in maternal livers.

**Fatty Acid (mg/100g)**	CD-vehicle (n = 5)	CD-DHA (n = 7)	HFD-vehicle (n = 6)	HFD-DHA (n = 7)	P_{Diet}	P_{DHA}	$P_{Diet\ x\ DHA}$
**SFAs**							
C12:0 Lauric acid	1.2 ± 0.5	1.7 ± 0.5	3.3 ± 1.1	3.9 ± 1.3	**0.037**	0.570	0.975
C14:0 Myristic acid	16.2 ± 2.1	25.5 ± 3.8	54.1 ± 10.6	49.0 ± 11.8	**0.002**	0.817	0.422
C16:0 Palmitic acid	1219.4 ± 104.6	1405.9 ± 123.6	2323.9 ± 515.2	2463.1 ± 489.8	**0.010**	0.675	0.951
C18:0 Stearic acid	606.2 ± 34.1	600.8 ± 107.5	1215.9 ± 553.1	970.1 ± 332.3	0.157	0.710	0.722
C20:0 Arachidic acid	4.0 ± 1.6	7.4 ± 2.4	19.5 ± 17.6	25.4 ± 7.8	0.099	0.638	0.902
C22:0 Behenic acid	3.4 ± 0.9	3.7 ± 0.6	1.7 ± 0.7	2.8 ± 0.5	0.061	0.303	0.552
C24:0 Lignoceric acid	3.9 ± 1.0	12.1 ± 7.0	18.0 ± 14.2	1.5 ± 0.6	0.827	0.610	0.136
∑ SFAs	1854.4 ± 135.9	2057.8 ± 188.4	3636.7 ± 1008.2	3516.0 ± 773.9	**0.024**	0.951	0.811
**MUFAs**							
C16:1 Palmitoleic acid	42.8 ± 12.8	93.0 ± 15.4	267.2 ± 155.0	145.3 ± 41.3	0.095	0.655	0.289
C18:1 Oleic acid	751.8 ± 90.3	967.7 ± 105.1	3087.7 ± 824.7	3396.4 ± 689.6	**<0.001**	0.647	0.935
C20:1 Eicosanoic acid	11.4 ± 1.6	12.8 ± 1.7	29.6 ± 6.4	50.7 ± 9.9	**<0.001**	0.108	0.136
C22:1 Erucic acid	3.6 ± 1.6	4.4 ± 1.2	5.7 ± 3.6	10.6 ± 2.4	0.101	0.252	0.389
C24:1 Nervonic acid	1.2 ± 1.2	1.8 ± 1.1	2.7 ± 0.9	0.01 ± 0.01	0.861	0.267	0.102
∑ MUFAs	813.4 ± 95.3	1082.8 ± 117.8	3394.9 ± 931.0	3610.5 ± 728.1	**0.001**	0.698	0.966
**PUFAs N-6**							
C18:2 Linoleic acid	1069.4 ± 66.8	1267.2 ± 123.1	2009.6 ± 517.5	1865.0 ± 386.2	**0.035**	0.939	0.622
C18:3 Gamma-linolenic acid	13.1 ± 7.6	11.9 ± 6.7	111.3 ± 52.5	31.5 ± 27.6	0.065	0.195	0.208
C20:2 Eicosadienoic acid	18.6 ± 2.0	21.0 ± 3.0	34.3 ± 8.7	38.2 ± 9.7	**0.033**	0.664	0.921
C20:3 Dihomo-γ-linolenic acid	23.3 ± 3.4	25.5 ± 2.1	31.0 ± 8.8	21.6 ± 5.7	0.739	0.530	0.320
C20:4 Arachidonic acid	500.5 ± 41.0	484.0 ± 30.0	624.4 ± 31.3	457.0 ± 86.5	0.403	0.120	0.198
∑ n-6 FAs	1624.9 ± 91.5	1809.7 ± 145.4	2810.5 ± 579.4	2413.4 ± 463.3	**0.034**	0.791	0.470
**PUFAs N-3**							
C18:3 Alfa-linolenic acid	22.5 ± 10.0	28.8 ± 12.5	62.9 ± 15.0	20.0 ± 8.4	0.199	0.140	0.052
C20:5 Eicosapentaenoic acid	22.2 ± 3.1	26.4 ± 3.1	35.9 ± 18.7	41.3 ± 8.3	0.185	0.654	0.958
C22:5 Docosapentaenoic acid	53.3 ± 3.5	58.0 ± 4.0	43.6 ± 8.1	34.3 ± 8.2	**0.021**	0.737	0.309
C22:6 Docosahexaenoic acid	752.3 ± 20.1	756.7 ± 48.1	687.6 ± 73.6	570.2 ± 100.0	0.100	0.448	0.414
∑ n-3 FAs	850.3 ± 12.4	869.9 ± 55.1	831.0 ± 107.3	665.7 ± 120.6	0.237	0.437	0.326
∑ PUFAs	2515.8 ± 78.5	2687.4 ± 186.9	3609.4 ± 642.2	3156.6 ± 599.0	0.115	0.770	0.518
C20:5/ C18:3 n-3	1.9 ± 0.6	1.6 ± 0.3	0.4 ± 0.2	5.3 ± 2.1	0.375	0.071	**0.047**
n-3/n-6 FAs	0.5 ± 0.02	0.5 ± 0.02	0.3 ± 0.1	0.3 ± 0.02	**<0.001**	0.162	0.711
18:3/18:2 n-6	0.02 ± 0.01	0.01 ± 0.00	0.05 ± 0.02	0.03 ± 0.01	**0.017**	0.342	0.575
20:4/20:3 n-6	24.1 ± 4.6	20.3 ± 2.4	27.6 ± 6.0	26.5 ± 7.4	0.400	0.666	0.816

Values are means ± SEM. Differences were calculated by two-way ANOVA followed Sidak's post-test.

Supplemental Table 4. Fatty acid profile in placentas from male fetuses.

**Fatty Acid (mg/100g)**	CD-vehicle (n = 5)	CD-DHA (n = 3)	HFD-vehicle (n = 5)	HFD-DHA (n = 5)	P_{Diet}	P_{DHA}	$P_{Diet\ x\ DHA}$
**SFAs**							
C12:0 Lauric acid	0.13 ± 0.06	ND	0.30 ± 0.11	0.21 ± 0.12	-	-	-
C14:0 Myristic acid	4.07 ± 0.57	3.11 ± 0.33	4.67 ± 1.15	3.89 ± 0.34	0.385	0.281	0.906
C16:0 Palmitic acid	177.9 ± 16.1	165.0 ± 18.6	182.6 ± 22.1	174.1 ± 9.9	0.702	0.556	0.904
C18:0 Stearic acid	234.5 ± 19.5	214.1 ± 16.9	253.9 ± 12.0	228.6 ± 6.9	0.271	0.144	0.870
C20:0 Arachidic acid	2.04 ± 0.41	1.79 ± 0.40	3.68 ± 1.78	1.34 ± 0.18	0.584	0.244	0.343
C22:0 Behenic acid	4.59 ± 0.34	2.82 ± 1.43	4.81 ± 0.25	4.65 ± 0.33	0.083	0.101	0.167
C24:0 Lignoceric acid	12.04 ± 1.11	12.28 ± 1.57	6.18 ± 0.95	6.72 ± 0.32	**<0.001**	0.702	0.883
∑ SFAs	435.2 ± 36.1	399.1 ± 33.2	456.1 ± 36.4	419.5 ± 16.7	0.540	0.286	0.994
**MUFAs**							
C16:1 Palmitoleic acid	6.67 ± 0.79	7.71 ± 1.66	5.75 ± 0.72	8.43 ± 0.83	0.918	0.068	0.397
C18:1 Oleic acid	28.36 ± 3.92	26.81 ± 4.08	52.78 ± 19.91	27.35 ± 6.84	0.333	0.296	0.353
C20:1 Eicosanoic acid	9.26 ± 3.38	6.18 ± 0.87	15.00 ± 4.99	10.35 ± 1.06	0.184	0.295	0.828
C22:1 Erucic acid	5.40 ± 0.79	5.70 ± 0.91	3.22 ± 1.13	2.64 ± 0.46	**0.011**	0.875	0.625
C24:1 Nervonic acid	ND	ND	ND	ND	**-**	**-**	**-**
∑ MUFAs	49.69 ± 6.72	46.40 ± 5.88	76.75 ± 18.54	48.77 ± 8.09	0.257	0.230	0.338
**PUFAs N-6**							
C18:2 Linoleic acid	56.43 ± 5.41	53.26 ± 4.19	49.82 ± 7.47	51.49 ± 4.34	0.498	0.903	0.694
C18:3 Gamma-linolenic acid	0.33 ± 0.13	0.33 ± 0.03	0.32 ± 0.13	0.42 ± 0.06	0.683	0.805	0.798
C20:2 Eicosadienoic acid	4.80 ± 4.80	ND	ND	ND	-	-	-
C20:3 Dihomo-γ-linolenic acid	14.30 ± 2.26	16.55 ± 1.42	7.99 ± 2.35	15.97 ± 1.19[b]	0.116	**0.026**	0.185
C20:4 Arachidonic acid	153.4 ± 17.4	142.8 ± 15.6	154.3 ± 22.2	160.8 ± 8.7	0.600	0.909	0.639
∑ n-6 FAs	229.2 ± 25.4	212.9 ± 19.3	212.5 ± 27.6	228.7 ± 13.5	0.984	0.997	0.506
**PUFAs N-3**							
C18:3 Alfa-linolenic acid	2.87 ± 0.24	3.18 ± 0.20	3.46 ± 0.23	3.70 ± 0.35	0.076	0.353	0.917
C20:5 Eicosapentaenoic acid	7.69 ± 1.00	8.64 ± 2.53	18.43 ± 2.06	19.82 ± 1.39	**< 0.001**	0.512	0.901
C22:5 Docosapentaenoic acid	11.12 ± 0.84	12.32 ± 0.46	10.28 ± 0.42	10.04 ± 0.36	**0.020**	0.433	0.246
C22:6 Docosahexaenoic acid	180.0 ± 17.9	179.4 ± 10.1	135.5 ± 17.6	153.6 ± 7.1	**0.038**	0.577	0.552
∑ n-3 FAs	201.7 ± 19.1	203.6 ± 11.3	167.7 ± 19.1	187.2 ± 8.0	0.153	0.532	0.605
∑ PUFAs	430.9 ± 44.4	416.5 ± 0	380.2 ± 0	415.9 ± 0	0.535	0.796	0.545
C20:5/ C18:3 n-3	2.77 ± 0.45	2.65 ± 0.83	5.38 ± 0.63	5.44 ± 0.37	**0.001**	0.784	0.864
n-3/n-6 FAs	1.89 ± 0.02	1.96 ± 0.66	1.80 ± 0.01	1.82 ± 0.02	0.341	0.217	0.177
18:3/18:2 n-6	0.005 ± 0.001	0.006 ± 0	0.006 ± 0.002	0.008 ± 0.001	0.366	0.578	0.904
20:4/20:3 n-6	13.73 ± 5.25	8.59 ± 0.24	41.17 ± 19.00	10.15 ± 0.37	0.223	0.135	0.275

Values are means ± SEM. Differences were calculated by two-way ANOVA following Sidak's post-test. [b]$P < 0.05$ between HFD-vehicle and HFD-DHA groups.

Supplemental Table 5. Fatty acid profile in placentas from female fetuses.

**Fatty Acid (mg/100g)**	CD-vehicle (n = 3)	CD-DHA (n = 5)	HFD-vehicle (n = 3)	HFD-DHA (n = 5)	P_{Diet}	P_{DHA}	$P_{Diet \times DHA}$
**SFAs**							
C12:0 Lauric acid	0.21 ± 0.01	ND	0.15 ± 0.15	0.09 ± 0.04	-	-	-
C14:0 Myristic acid	3.24 ± 0.31	4.44 ± 0.71	3.84 ± 0.28	3.26 ± 0.22	0.583	0.560	0.113
C16:0 Palmitic acid	163.5 ± 9.5	200.8 ± 21.9	189.1 ± 16.2	160.0 ± 9.6	0.669	0.816	0.080
C18:0 Stearic acid	230.6 ± 6.6	253.8 ± 25.5	246.3 ± 22.6	224.0 ± 10.9	0.735	0.982	0.286
C20:0 Arachidic acid	1.58 ± 0.29	3.14 ± 0.75	1.49 ± 0.16	1.25 ± 0.1	0.080	0.222	0.107
C22:0 Behenic acid	4.89 ± 0.24	4.77 ± 0.43	4.88 ± 0.49	4.65 ± 0.21	0.858	0.657	0.882
C24:0 Lignoceric acid	10.51 ± 1.1	13.51 ± 0.61	7.18 ± 0.28	5.53 ± 0.7	**<0.0001**	0.378	**0.009**
∑ SFAs	414.5 ± 5.6	480.4 ± 49.6	452.9 ± 38.6	398.7 ± 18.6	0.580	0.879	0.140
**MUFAs**							
C16:1 Palmitoleic acid	7.04 ± 1.12	5.25 ± 0.43	9.99 ± 1.34	6.51 ± 1.34	0.089	**0.039**	0.471
C18:1 Oleic acid	24.09 ± 3.52	32.79 ± 4.02	34.48 ± 3.49	54.34 ± 25.17	0.368	0.420	0.750
C20:1 Eicosanoic acid	5.32 ± 0.45	15.16 ± 3.78	10.56 ± 1.3	8.63 ± 0.68	0.808	0.155	**0.043**
C22:1 Erucic acid	4.8 ± 0.54	4.54 ± 0.86	1.98 ± 0.1	2.07 ± 0.18	**0.001**	0.892	0.779
C24:1 Nervonic acid	ND	ND	ND	ND	**-**	**-**	**-**
∑ MUFAs	41.25 ± 5.23	57.74 ± 7.21	57.01 ± 6.06	71.56 ± 24.46	0.408	0.386	0.956
**PUFAs N-6**							
C18:2 Linoleic acid	49.78 ± 4.74	61.97 ± 7.16	57.78 ± 5.61	45.87 ± 3.85	0.516	0.983	0.070
C18:3 Gamma-linolenic acid	0.28 ± 0.03	0.68 ± 0.12	0.37 ± 0.14	0.43 ± 0.11	0.527	0.073	0.175
C20:2 Eicosadienoic acid	ND	4.91 ± 4.91	3.8 ± 1.99	ND	-	-	-
C20:3 Dihomo-γ-linolenic acid	15.14 ± 1.36	10.69 ± 2.2	17.71 ± 1.69	14.4 ± 1.28	0.120	0.061	0.766
C20:4 Arachidonic acid	138.9 ± 16.0	183.4 ± 20.4	184.8 ± 18.6	146.9 ± 10.7	0.798	0.856	**0.039**
∑ n-6 FAs	204.1 ± 21.6	261.7 ± 27.0	264.5 ± 25.7	207.6 ± 15.6	0.899	0.989	**0.035**
**PUFAs N-3**							
C18:3 Alfa-linolenic acid	2.95 ± 0.06	3.47 ± 0.13	3.66 ± 0.33	3.27 ± 0.09	0.139	0.707	**0.016**
C20:5 Eicosapentaenoic acid	9.11 ± 1.79	11.21 ± 2.23	25.22 ± 3.67	18.78 ± 1.61	**< 0.001**	0.377	0.096
C22:5 Docosapentaenoic acid	11.29 ± 0.61	11.77 ± 0.59	10.57 ± 0.59	9.79 ± 0.24	**0.025**	0.772	0.253
C22:6 Docosahexaenoic acid	157.0 ± 13.8	212.8 ± 21.6	162.7 ± 13.5	137.6 ± 11.1	0.072	0.402	**0.041**
∑ n-3 FAs	180.4 ± 11.1	239.2 ± 20.9	202.1 ± 14.8	169.4 ± 12.5	0.203	0.477	**0.025**
∑ PUFAs	384.5 ± 35.5	500.9 ± 47.8	466.6 ± 40.4	377.0 ± 28.0	0.626	0.754	**0.030**
C20:5/ C18:3 n-3	3.11 ± 0.66	3.23 ± 0.63	6.87 ± 0.67	5.75 ± 0.45	**< 0.001**	0.438	0.333
n-3/n-6 FAs	1.89 ± 0.03	1.92 ± 0.02	1.77 ± 0.02	1.82 ± 0.01	**< 0.001**	0.058	0.674
18:3/18:2 n-6	0.01 ± 0	0.01 ± 0	0.01 ± 0	0.01 ± 0	0.897	0.080	0.732
20:4/20:3 n-6	9.13 ± 0.25	21.27 ± 5.17	10.44 ± 0.53	10.26 ± 0.28	0.187	0.110	0.100

Values are means ± SEM. Differences were calculated by two-way ANOVA followed Sidak's post-test.

Supplemental Table 6. Fatty acid profile in liver from male fetuses.

**Fatty Acid (mg/100g)**	CD-vehicle (n = 5)	CD-DHA (n = 3)	HFD-vehicle (n = 6)	HFD-DHA (n = 4)	P_{Diet}	P_{DHA}	$P_{Diet \times DHA}$
**SFAs**							
C12:0 Lauric acid	0.001 ± 0.001	ND	0.01 ± 0.001	0.002 ± 0.001	-	-	-
C14:0 Myristic acid	0.08 ± 0	0.08 ± 0.01	0.12 ± 0.01	0.08 ± 0.02	0.207	0.154	0.224
C16:0 Palmitic acid	3.29 ± 0.14	3.17 ± 0.19	3.67 ± 0.23	2.59 ± 0.76	0.799	0.150	0.250
C18:0 Stearic acid	2.28 ± 0.06	2.3 ± 0.03	2.19 ± 0.08	1.69 ± 0.43	0.109	0.266	0.230
C20:0 Arachidic acid	0.04 ± 0.01	0.03 ± 0	0.05 ± 0.01	0.03 ± 0.01	0.452	0.064	0.507
C22:0 Behenic acid	0.03 ± 0	0.03 ± 0	0.03 ± 0	0.02 ± 0.01	0.136	**0.017**	0.608
C24:0 Lignoceric acid	0.06 ± 0.01	0.04 ± 0.02	0.03 ± 0	0.02 ± 0.01	**0.008**	0.072	0.651
∑ SFAs	5.79 ± 0.17	5.64 ± 0.15	6.09 ± 0.32	4.43 ± 1.23	0.465	0.158	0.236
**MUFAs**							
C16:1 Palmitoleic acid	0.41 ± 0.05	0.29 ± 0.07	0.64 ± 0.06	0.22 ± 0.1[b]	0.303	**0.002**	0.055
C18:1 Oleic acid	0.69 ± 0.02	0.74 ± 0.06	0.81 ± 0.05	0.38 ± 0.2	0.241	0.080	**0.032**
C20:1 Eicosanoic acid	0.11 ± 0.02	0.09 ± 0.01	0.12 ± 0.03	0.06 ± 0.02	0.742	0.150	0.455
C22:1 Erucic acid	0.04 ± 0.01	0.03 ± 0.01	0.03 ± 0.01	0.03 ± 0.01	0.606	0.550	0.763
C24:1 Nervonic acid	ND	ND	ND	ND	-	-	-
∑ MUFAs	1.25 ± 0.07	1.15 ± 0.03	1.60 ± 0.12	0.68 ± 0.3[b]	0.727	**0.009**	**0.026**
**PUFAs N-6**							
C18:2 Linoleic acid	1.10 ± 0.07	0.89 ± 0.09	1.07 ± 0.09	0.81 ± 0.25	0.712	0.118	0.847
C18:3 Gamma-linolenic acid	0.05 ± 0.02	0.05 ± 0.02	0.02 ± 0	0 ± 0	**0.010**	0.644	0.631
C20:2 Eicosadienoic acid	0.06 ± 0.04	ND	0.04 ± 0.03	ND	-	-	-
C20:3 Dihomo-γ-linolenic acid	0.12 ± 0.01	0.11 ± 0.01	0.09 ± 0.02	0.09 ± 0.03	0.219	0.825	0.933
C20:4 Arachidonic acid	1.56 ± 0.07	1.38 ± 0.01	1.54 ± 0.09	0.84 ± 0.46	0.229	0.067	0.259
∑ n-6 FAs	2.88 ± 0.12	2.43 ± 0.11	2.76 ± 0.18	1.75 ± 0.69	0.248	0.069	0.485
**PUFAs N-3**							
C18:3 Alfa-linolenic acid	0.08 ± 0.06	0.01 ± 0	0.02 ± 0	0.01 ± 0	0.474	0.336	0.455
C20:5 Eicosapentaenoic acid	0.06 ± 0.01	0.07 ± 0.03	0.17 ± 0.02	0.11 ± 0.03	**0.017**	0.395	0.252
C22:5 Docosapentaenoic acid	0.04 ± 0	0.05 ± 0.01	0.04 ± 0.00	0.03 ± 0.01	0.068	0.946	0.234
C22:6 Docosahexaenoic acid	3.12 ± 0.23	2.73 ± 0.27	2.09 ± 0.09	1.76 ± 0.55	**0.005**	0.253	0.922
∑ n-3 FAs	3.30 ± 0.27	2.87 ± 0.22	2.32 ± 0.10	1.92 ± 0.59	**0.012**	0.274	0.900
∑ PUFAs	6.18 ± 0.38	5.3 ± 0.33	5.08 ± 0.25	3.66 ± 1.23	**0.049**	0.091	0.681
EPA/ALA	6.80 ± 4.63	7.64 ± 5.35	7.89 ± 1.55	8.29 ± 1.05	0.803	0.859	0.951
n-3/n-6 FAs	1.14 ± 0.05	1.18 ± 0.05	0.84 ± 0.02	1.24 ± 0.34	0.472	0.199	0.281
C18:3/18:2 n-6	0.04 ± 0.01	0.05 ± 0.02	0.02 ± 0.01	0 ± 0	**0.007**	0.825	0.388
20:4/20:3 n-6	13.28 ± 0.84	12.30 ± 0.88	30.05 ± 12.67	8.99 ± 3.18	0.475	0.250	0.292

Values are means ± SEM. Differences were calculated by two-way ANOVA followed Sidak's post-test. [b]$P < 0.05$ between HFD-vehicle and HFD-DHA groups.

Supplemental Table 7. Fatty acid profile in liver from female fetuses.

**Fatty Acid (mg/100g)**	CD-vehicle (n = 3)	CD-DHA (n = 4)	HFD-vehicle (n = 3)	HFD-DHA (n = 5)	***P_{Diet}***	***P_{DHA}***	***$P_{Diet\ x\ DHA}$***
**SFAs**							
C12:0 Lauric acid	0.003 ± 0.002	0.003 ± 0.002	ND	0.003 ± 0.002	-	-	-
C14:0 Myristic acid	0.08 ± 0.01	0.09 ± 0.01	0.11 ± 0.02	0.09 ± 0.02	0.402	0.472	0.829
C16:0 Palmitic acid	3.15 ± 0.41	3.4 ± 0.32	3.4 ± 0.39	2.85 ± 0.64	0.785	0.469	0.790
C18:0 Stearic acid	2.27 ± 0.24	2.19 ± 0.14	2.07 ± 0.15	1.87 ± 0.42	0.450	0.853	0.670
C20:0 Arachidic acid	0.04 ± 0.01	0.05 ± 0	0.04 ± 0.03	0.03 ± 0.01	0.364	0.658	0.970
C22:0 Behenic acid	0.03 ± 0	0.02 ± 0.01	0.03 ± 0	0.02 ± 0	0.815	0.597	0.065
C24:0 Lignoceric acid	0.06 ± 0.01	0.03 ± 0.02	0.03 ± 0	0.02 ± 0.01	0.067	0.224	0.072
∑ SFAs	5.63 ± 0.65	5.77 ± 0.46	5.67 ± 0.57	4.88 ± 1.09	0.640	0.609	0.720
**MUFAs**							
C16:1 Palmitoleic acid	0.4 ± 0.08	0.49 ± 0.06	0.61 ± 0.13	0.4 ± 0.14	0.620	0.220	0.630
C18:1 Oleic acid	0.69 ± 0.09	0.73 ± 0.07	0.8 ± 0.12	0.67 ± 0.15	0.863	0.512	0.753
C20:1 Eicosanoic acid	0.1 ± 0.03	0.05 ± 0.02	0.09 ± 0.01	0.08 ± 0.02	0.645	0.396	0.191
C22:1 Erucic acid	0.05 ± 0.02	0.08 ± 0.01	0.04 ± 0.01	0.03 ± 0.01	**0.036**	0.147	0.315
C24:1 Nervonic acid	ND	ND	ND	ND	**-**	**-**	**-**
∑ MUFAs	1.23 ± 0.18	1.35 ± 0.11	1.53 ± 0.28	1.18 ± 0.29	0.799	0.360	0.653
**PUFAs N-6**							
C18:2 Linoleic acid	1.06 ± 0.13	0.7 ± 0.25	1.1 ± 0.19	0.82 ± 0.2	0.699	0.854	0.167
C18:3 Gamma-linolenic acid	0.03 ± 0.02	0 ± 0	0.01 ± 0	0.01 ± 0	0.329	0.139	0.146
C20:2 Eicosadienoic acid	0.05 ± 0.05	ND	0.01 ± 0.01	ND	-	-	-
C20:3 Dihomo-γ-linolenic acid	0.13 ± 0.01	0.14 ± 0.01	0.12 ± 0.01	0.1 ± 0.02	0.270	0.514	0.787
C20:4 Arachidonic acid	1.49 ± 0.19	1.48 ± 0.19	1.09 ± 0.54	1.23 ± 0.31	0.346	0.819	0.845
∑ n-6 FAs	2.75 ± 0.33	2.31 ± 0.33	2.33 ± 0.73	2.16 ± 0.73	0.590	0.796	0.573
**PUFAs N-3**							
C18:3 Alfa-linolenic acid	0.02 ± 0	0.02 ± 0.01	0.03 ± 0.01	0.03 ± 0.01	0.347	0.801	0.839
C20:5 Eicosapentaenoic acid	0.07 ± 0.03	0.09 ± 0.02	0.17 ± 0.01	0.11 ± 0.02	**0.013**	0.086	0.313
C22:5 Docosapentaenoic acid	0.04 ± 0	0.04 ± 0.02	0.04 ± 0	0.03 ± 0.01	0.573	0.425	0.435
C22:6 Docosahexaenoic acid	3.00 ± 0.48	2.79 ± 0.34	2.06 ± 0.24	1.77 ± 0.42	**0.035**	0.924	0.554
∑ n-3 FAs	3.13 ± 0.49	2.94 ± 0.35	2.3 ± 0.26	1.93 ± 0.45	0.057	0.843	0.527
∑ PUFAs	5.89 ± 0.81	5.25 ± 0.66	4.63 ± 0.99	4.1 ± 0.97	0.220	0.958	0.541
EPA/ALA	3.23 ± 0.84	4.45 ± 1.09	7.54 ± 1.45	5.24 ± 1.03	0.053	0.160	0.650
n-3/n-6 FAs	1.13 ± 0.06	1.29 ± 0.11	1.25 ± 0.41	1.13 ± 0.25	0.931	0.583	0.927
18:3/18:2 n-6	0.02 ± 0.02	0 ± 0	0.01 ± 0	0.02 ± 0.02	0.940	0.221	0.876
20:4/20:3 n-6	11.57 ± 0.97	10.8 ± 0.84	8.52 ± 4.28	9.92 ± 2.57	0.457	0.679	0.904

Values are means ± SEM. Differences were calculated by two-way ANOVA followed Sidak's post-test.
