# Supplementary material for: Effects of a High-Fat Diet and Docosahexaenoic Acid during Pregnancy on Fatty Acid Composition in the Fetal Livers of Mice
Source: Nutrients. 2023 Nov 6;15(21):4696. doi: 10.3390/nu15214696 (PMC10649644; doi:10.3390/nu15214696)
Supplement: Supplementary file 1 [file nutrients-15-04696-s001.zip › nutrients-2673161-supplementary/Suplemmentary material/Supplementary Figure 1.pdf]

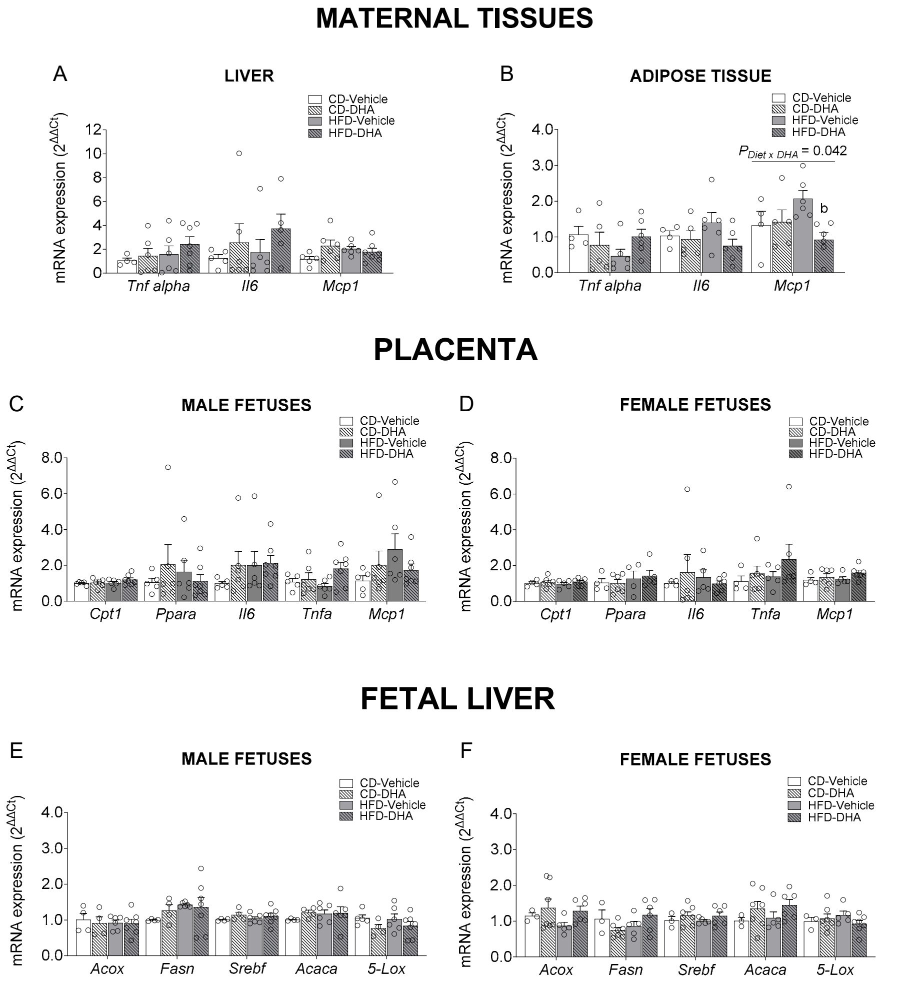

Supplementary Figure 1. Gene expression (mRNA) in maternal tissues, placentas and fetal liver of male and female fetuses from dams fed with a control diet (CD), high-fat diet (HFD), and treated with vehicle (sunflower oil) or docosahexaenoic acid (DHA) during pregnancy. (A and B) Gene expression of tumoral necrosis factor alpha (*Tnfa*), interleukin-6 (*Il6*), and monocyte chemoattractant protein-1 (*Mcp1*) in the maternal liver and adipose tissue. (C - D) carnitine palmitoyltransferase I (*Cpt1*), peroxisome proliferator-activated receptor alpha (*Ppara*), *Il6*, *Tnfa,* and *Mcp1* in placental tissue from male and female fetuses. (E – F) Gene expression of Acyl-CoA oxidase (*Acox*), fatty acid synthase (*Fasn*), sterol regulatory element binding transcription factor (*Srebf*), acetyl-CoA carboxylase alpha (*Acaca*), and 5-lipoxygenase (*5-Lox*) in the liver of male and female fetuses. Values are means ± SEM. Two-way ANOVA followed Sidak's post-test was performed to calculate the differences.
